# Hospital Practice

**Published:** 1828-04-01

**Authors:** 


### HOSPITAL PRACTICE.

Under this head, we hope to give a selection and re-union of important cases reported from the public institutions of this and other countries, which will prove the most valuable department of a journal ever offered to the medical practitioner, of town or country. We shall take great care to disembarrass reports of all useless or irrelevant verbiage—nor shall we notice those cases that afford no useful practical hints, or interesting phenomena. In our criticisms, we shall be guided solely by rigid impartiality—and a desire to correct error and establish truth.

### 1. ST. GEORGE'S HOSPITAL.

#### STATE OF THE PUPIL IN DEEP INTOXICATION.*

A young man was brought into this hospital, some little time ago, having fallen from the Bristol coach half an hour previously, in a state of complete intoxication. When admitted, he was quite insensible—the pulse 70, and rather labouring—the pupil dilated at first, but, in the course of a very short time, it had become contracted almost to a pin's point. At the back of the head there was a circular wound of the scalp, about the size of a shilling, exposing the bone perfectly denuded beneath, but unaccompanied with fracture or depression. In the course of the day the pulse got up, and he was bled; and in the evening he brought up, by vomiting, a most disgusting mass of pork, brandy, &c. after which he became much more sensible, and the pupils began to act. Next day he complained of nothing but the scalp-wound, in spite of which, and all Mr. Rose's remonstrances to boot, he walked out of the hospital, got upon the top of the coach, and set off once more for Bristol.

Upon this case the reporter remarks—"We mention it principally with a view of calling the attention of our readers to the excessive contraction of the pupil, a state which Mr. Rose says he has generally seen in complete intoxication. In this patient there were many of the symptoms of compression of the brain; and indeed there cannot be a doubt that individuals are occasionally most severely, and even fatally treated for such compression, when rest, or, it may be, the stomach-pump, are all that is wanted."

* London Medical Gazette, No. V

#### STRANGULATED HERNIA.

Two cases are reported in the Medical Gazette, (No. 4,) which we shall notice, in order to shew Mr. Brodie's practice.

The first case was that of a stout young countryman, admitted under the care of Mr. Rose. The hernia had existed since childhood, was never completely reducible, and the symptoms of strangulation had obtained for twenty-four hours prior to the performance of the operation. On opening the sac, a knuckle of intestine was found, which was returned, but, besides this, there remained a quantity of omentum, which had contracted old and strong adhesions to the sac. This was left unreduced; an abscess subsequently formed in it, attended with pretty urgent symptoms, but under the judicious treatment of Mr. Rose the patient did remarkably well.

The second case occurred to Mr. Brodie. The patient was a watchman—the symptoms commenced at 5, A.M. of the 15th December, and the operation was performed at 3, A.M. of the 16th. As in the former instance, intestine and omentum were down, and the circumstances being the same, the intestine was returned, and the omentum left undisturbed in the sac. This patient also did well.

Surgeons are now so universally impressed with the advantages of the early operation in hernia, that we shall say not a word about that. The treatment of the omentum in these cases is the point to which we would wish to direct the attention of our readers. Sir Astley Cooper, in his published lectures, (and a greater boon than these lectures, we think, has not been conferred on the rising professional generation for these many years,) makes the following remarks. If the omentum be very large—mortified—or, if it be "hardened and have a scirrhous feel," it should be removed wholly, or in part, the divided vessels secured by fine ligatures, and these, one end being cut off, should be left hanging from the wound. If omentum alone adheres to the sac, *it may be freely separated and returned.* So far, Sir Astley; Mr. Brodie, however, thinks otherwise, and as the reporter has detailed his method of treatment, we shall take the liberty of quoting the passage, without note or comment of our own.

"In his (Mr. Brodie's) Clinical Lecture upon Joseph Haplin's case, he observed, that whenever difficulty obtained in reducing the omentum, either from its being adherent, mortified, or from its being converted into a large fatty mass, the old practice used to be to cut it off, or apply a ligature upon it. With regard to the ligature, authors are almost unanimous in disapproving of it; in fact, as Sir A. Cooper shrewdly remarks, it is renewing with increased severity what the operation was intended to relieve—the stricture on the part.

"Mr. Brodie is inclined to think that the excision of the omentum is not so devoid of difficulty and danger as some would seem to infer. If no vessels are tied, the hemorrhage may be very considerable; indeed, Mr. Hey has published two cases in which the patients nearly died of it. If, again, the arteries are tied, the ligatures cut off close, and the omentum returned, we are not sure that bleeding may not take place within the abdomen, from vessels which did not bleed while the omentum lay exposed in the sac, and which therefore were not secured: and, besides, what is to become of the ligatures? Mr. Earle related to Mr. Brodie a case of this kind, in which they were discharged through an abscess which presented itself at or near the umbilicus. The patient recovered; but, nevertheless, an abscess of the belly, which bursts at the umbilicus, cannot be supposed to be always free from danger. If, instead of the ligatures being cut close, one end of each of them be left hanging out at the neck of the hernial sac, the omentum must of course remain drawn down to the abdominal ring; and in what respect is the patient better off than if a portion of it had been left in the sac itself? There is also some danger in this case of inflammation of the cut end of the omentum, and Mr. Brodie mentioned one case in which an abscess formed in the omentum, at this part, immediately within the internal abdominal ring, and the patient died in consequence.

"He, however, stated that he did not mean to condemn the excision of the omentum in all cases, but merely to express that it ought not to be done without a very sufficient reason; and that in cases where there is not a very large quantity of omentum in the hernia, but where it is extensively adherent to the surface of the sac, it is safer to leave it where it is found, than to cut off a portion of it, or to dissect through the adhesions. The success of this practice, in the two preceding cases, is certainly in its favour. The patients are left as well off as they were before the strangulation took place; and probably better, inasmuch as it is most likely that the omentum, after the operation, must have contracted adhesions to the neck of the hernial sac, making them less liable to a descent of the intestine."

---

### 2. MIDDLESEX HOSPITAL.

### ANOMALOUS ŒDEMATODE SWELLING OF THE LEG.*

A watchman, æt. 32, was admitted in October, with an enlargement in the left leg and thigh, which had begun suddenly, as we understand it, three years previously, had never diminished, except when in the recumbent position, and was productive of much distress and suffering. His constitution was broken down—secretions imperfect, and he voided with much difficulty turbid and scanty urine. The left leg was about 22 inches in circumference, natural in colour, tender to the touch, elastic, but indenting on firm pressure. He took diuretics, was cupped and blistered for the first three weeks after his admission, when an acute attack of hepatitis came on, which was subdued only by the most active treatment. During the attack, the leg was free from pain, and had nearly regained its natural size, but the moment the hepatic inflammation ceased, the swelling, &c. of the leg returned. This happened a second time in November, on the 25th of which he left the hospital, in consequence of some expressions inadvertently used, and has never since returned. The reporter

* London Med. Gazette, No. V.

observes, that this case bears some resemblance to phlegmatia dolens, and in a female, after parturition, might have been so considered. The symptoms, however, of phlegmatia dolens do not remain unrelieved for so long a period as three years, though, certainly a degree of swelling of the limb may remain for life. To us, it appears that the case was one of chronic inflammation of the cellular tissue generally, and this opinion is strengthened by the fact of its alternating with the acute inflammation of the liver. We have seen one or two cases, somewhat similar, occurring in patients who were much exposed to damp and cold, and where the appearance of the limb was far from being unlike that presented in phlegmatia dolens, although the disease ran on to a different and fatal termination.

### 3. ST. THOMAS'S HOSPITAL.

ABSCESS BETWEEN THE BLADDER AND PUBES.

This case is related in the fifth number of the Gazette. The patient was a female, æt. 42, who had been married at the age of 20, and miscarried in the sixth month. She was not pregnant again for 18 years, when she again miscarried in the fifth month. After this she did not menstruate for 18 months, when she was attacked by profuse flooding, which lasted for three days. In the following month she began to suffer from pain in the uterine and lumbar regions, and November 21st she entered the hospital under the care of Dr. Elliotson. She was sallow, debilitated, and complained of violent pain in the hypogastrium, pubes, and loins, with numbness and tingling in the right thigh. Tenderness of the hypogastrium and pubes to the touch—copious discharge of puriform matter from the vagina, occasionally tinged with blood—urine generally free, sometimes retained for two days together—general ill health. The uterus, on examination, was healthy, but the discharge dark and fetid. These symptoms were not relieved by calomel, leeches, blisters, &c. &c.—the discharge continued copious—cough came on—she grew weaker, and on the 16th December she sank.

*On examination*, there was found in front and behind the symphysis, extending beyond the external abdominal rings, so that the round ligaments passed through it, an abscess, containing dark and fetid pus, which had a free exit through the urethra. A portion of this was destroyed —the surface of the bone was rough and blackened, but not carious, whilst the bladder nnd vagina were perfectly healthy.

STRANGULATED HERNIA.

A case of strangulated hernia, terminating fatally, is reported in the last Number of the Lancet, which we deem it right to notice. The patient was a stout man, who had laboured under constipation of the bowels for some days, attended with pain and colic, for which, in fact, he was sent into the hospital, no allusion being made to hernia, which, however, was speedily discovered there. The swelling took the usual course of inguinal hernia—occupied a portion of scrotum—and the integuments were inflamed. The man had vomiting and hiccup—the countenance was not depressed—pulse feeble—much tenderness of abdomen. He had, according to his own account, a swelling in the part since his birth, but latterly it got larger. The taxis was ineffectually employed, and then Mr. Green proceeded to operate. The hernial sac contained much serum, and a knuckle of intestine, of a chocolate colour, was found, partially adherent to the sac. The hernia was of the congenital kind. Although the ring was freely divided, a stricture still existed, beyond the ring, girting the intestine. This being cut through, the gut was readily returned. It is the practice of Mr. Green not to give any purgative medicine for some hours after an operation for hernia, and this practice was followed in the present instance. The reporter, however, seems to have discovered a mighty flaw in the GAZETTE account of the case—namely, the administration of a couple of glysters *immediately after* the operation, instead of some sulphate of magnesia the next day at noon! Most important mis-statement! This beats the Lancet hollow. That veracious instrument never committed such an error, wilfully or accidentally! But, allowing that the enemata had been administered immediately after the operation, it would not have been an infraction of the precept laid down by Cline, and followed by Green—that of not disturbing the bowels too soon by purgative medicine. The enema merely empties the colon, and does not affect the small intestine. But this is a piece of knowledge far beyond the scope of the reporter's sensorium. The patient died in three days after the operation, and no dissection was permitted. This case is spun out with the most tiresome verbiage in the Lancet, to the extent of four columns! Surely some of the reporters must be paid by the *acre* rather than by the *sheet* of letter-press which they blacken in every volume of the LANCET!

---

#### 4. ST. BARTHOLOMEW'S HOSPITAL.

FRACTURE OF THE FOURTH AND FIFTH CERVICAL VERTEBRÆ.*

We cannot but congratulate our contemporary on his HOSPITAL REPORTS. They seem to be given in a spirit of great candour and fairness, and the cases are generally interesting, whilst they are unalloyed by that sickening ribaldry and slang which grace this department of the Lancet. This is as it should be.

*Case.* G. F. æt. 45, was admitted under the care of Mr. Vincent, at 8, A.M. Dec 18th, having fallen on the back of his head from the top of a loaded waggon, by which he was stunned for the time. On admission, the occipital bone was found denuded of its pericranium for some extent—pulse small and feeble—sensibility and motion diminished in the upper and lower extremities—pupils contracted—priapism—respiration chiefly diaphragmatic. When Mr. V. saw him at half-past twelve, there was complete paralysis of the lower half of the body, with pain in the back of the neck, where a projection could be felt, in the situation of the fifth cervical vertebra. The pulse rose, and at 12, P.M. he was bled to ℥xvij. Next day, the paralysis of the upper extremities had increased; he passed his stools involuntarily, and the bladder required the introduction of the catheter night and morning. He was bled and purged, but without any amendment, and on the 21st he died.

DISSECTION. The base of the spinous process of the fourth cervical vertebra was fractured, and its right articulating process thrown forwards on the fifth, the body of which was completely fractured through at its upper part. Blood was effused between the vertebræ and the theca, which was not torn, whilst the chord in the situation of the fracture was much swollen and softened, but not lacerated. Slight vascularity about the neck of the bladder.

Of what use would trephining have been here?

CASE 2.—*Morbid Growth in the Femur.* The patient was a wretched-looking creature, about 50, a martyr to rheumatic gout. The disease in the knee was of three years' standing, and had been once almost cured by an issue. The joint was greatly swollen, and projected in a conical form towards the inside of the leg. The apex appeared to contain fluid, but the rest of the tumour was firm and unyielding, like a fungous growth. There was excessive pain and constitutional disturbance; and, besides this, the poor wretch had diseased bladder and stricture of the urethra, so that he was no very promising subject for an operation. This was done by Mr. Vincent on a Saturday, and, on the succeeding Friday, the patient had a fit of the gout, and died.

On examining the tumour, much chalky substance was found in the subcutaneous cellular tissue, and on the head of the femur, the internal condyle of which was filled with a gelatinous, fungoid substance. It was something like soft soap, had broken down the bony walls of the condyle, and made its way into the joint and its neighbourhood.

It is not improbable, from the chalky deposition in the joint and around it, that this anomalous disease had its origin in the gouty or rheumatic inflammation.

HERNIA—ADHERENT EPIPLOCELE.

The Lancet, for reasons best known to itself, though, perhaps, obvious enough to *some* others, has hunted back to the month of November, 1826, for a case exemplifying certain points of practice, lately reported from St. George's Hospital, in a cotemporary journal. The object seems to be, to shew that Mr. Lawrence is not exactly of Mr. Brodie's way of thinking,

---

* London Med. Gaz. No. V.

in regard to the treatment of adherent omentum. Non nobis inter vos, &c.

The patient was 60 years of age, and had been ruptured for the last thirty. He had worn a truss, though the reduction was only partial. After some unusual exertion, on the 2d of November, a further protrusion occurred—the parts became painful—a surgeon tried ineffectually to replace the rupture—and he was sent into Bartholomew's. He evidently had strangulated hernia, and all the usual means were pursued, (and minutely detailed, for the ten-thousandth time,) without success. Mr. Lawrence operated at the expiration of eight hours from the new descent. Every stroke of the scalpel is, of course, described—but we shall inform our readers, at once, that six inches of incarcerated small intestine were found, of a dark chocolate colour. The stricture having been divided, an indentation (most wonderful to relate) was found in the strictured gut—and when this was drawn down, a small opening was found in the intestine, just above the indentation. "Mr. Lawrence supposed that the bowel had been wounded by the curved bistoury." He tied a silk thread round the aperture—cut away the ends—dissected away a portion of old, adherent omentum—tied the vessels—and the whole was then dressed lightly. The reporter of this case (which occupies four deadly columns of the Lancet) does not say whether the intestine was returned into the abdomen after ligature of the perforation. The patient recovered without any bad symptoms. Under the head of "REMARKS," the reporter, who seems to have an eye to the "main chance," in the *length* of his cases, as well as his *cuts*, repeats the whole business over again, with one or two quotations from Hey, Desault, Beyer, (a *new* name in the surgical horizon) Lawrence, and Travers. The whole case is unquestionably taken from the *porte-feuille* of a *man of letters*, for a special purpose, which we need not explain.

If ever a critical reviewer and condenser was needed in medical literature, it is at the present moment, for hospital reports. The writers are paid by the sheet—and it is their interest to *dilate*—the critical condenser is paid in a diametrically opposite manner—and the consequences are obvious.

### 5. GUY'S HOSPITAL.

LACERATION OF THE URETHRA—SLOUGHING—FATAL RESULT.*

W. S. æt. 14, was admitted, Nov. 19th, under the care of Mr. Key, having fallen on the perinæum across an iron chain. Severe pain and inability to make water, followed the accident, and, in half an hour, the scrotum began to swell, and became red. On admission, the scrotum was greatly distended and ecchymosed on its posterior part, with slight effusion into the prepuce.

*Scarifications into the scrotum—fomentations—catheter to be kept in the bladder.*

*22d.* Had an exacerbation of fever last night, but it is mitigated this morning—ecchymosed part of the scrotum livid—cellular membrane, which has been scarified, dark and sloughy—yellow hue on the lower part of the abdomen.

*Quin. sulph.* gr. jss. *Camph.* gr. ij. *Amm. carb.* gr. x. *6tis horis—Cat. cerevisiæ.*

Hiccough and tenderness of the hypogastrium came on, and this was so severe on the *24th* that, to prevent infiltration, it was determined to cut into the urethra, pass a catheter through the opening, and confine it in the bladder. On doing this, it was found that two inches of the urethra, anterior to the triangular ligament, had sloughed. Bowels relaxed—pulse 130 and weak.

*Tinct. opii,* ♏ x. *ex Julep. ammon.—Arrow-root and brandy.—Quinine to be omitted.*

A slough formed at the back of the scrotum, which separated and exposed the tunica vaginalis of the right testicle. On the *6th Dec.* an incision was made over Poupart's ligament, and a quantity of fetid pus discharged, but, on the *15th*, the boy died. No dissection was permitted.

Mr. Key, in his remarks on this case, observed that, in common cases of ruptured urethra, leaving a catheter in the bladder generally suffices, the inflammation arising from the extravasation generally setting bounds to the escape of the urine beyond the cells of the scrotum. In this case, the infiltration was exceedingly diffused, and free incisions into the

* London Med. Gazette, No. V.

scrotum did not prevent the occurrence of extensive sloughing, which, however, was the immediate consequence of the blow, and not of the extravasation. On the 24*th*, when the pain and redness about the pubes indicated still further extravasation, a free incision in perineo was resorted to, although Mr. Key feared, at the time, that the mischief proceeded more from the sloughing of the parts about the urethra, than from impediment to the exit of the urine. This opinion was unfortunately correct, for the infiltration extended between the muscles and peritoneum as high, nearly, as the diaphragm, shewing that the fascia which forms the boundary between the perineum and cellular tissue of the pelvis had sloughed. Under these circumstances, it was deemed proper to make a free opening for any matter which might form, and support the powers of the constitution. This was all that could be be done, and this was of no avail.

The case is reported in a very creditable manner.

---

### FATAL DISEASE OF THE BRAIN MISTAKEN.

A Gentleman (Mr. B.) aged about 30 years, had a fall from his horse, about the month of December, 1826, by which he was stunned, but from which he soon revived, and took no farther notice of the accident. In February of 1827, he began, for the first time, to feel deep-seated pains in his head, especially towards the occiput, and, soon afterwards, sickness at stomach, and even vomiting. These complaints gradually increased, and resisted every remedy. In the beginning of September he came up from the country to London, and went to a celebrated and eccentric surgeon. He was then very ill, and his wife told the surgeon that her husband was dreadfully afflicted with pain in his head, and sickness at his stomach. "say no more," said the surgeon—"I see what is the matter. Put out your tongue. Take this prescription, (giving him blue-pill at night, and black-draught in the morning,) and go home." He would not hear a single word of the patient's history, nor examine into a single symptom except the state of the tongue. In a week, the gentleman was much worse, and the writer of this article was sent for. The countenance of the patient, the vacant stare of the eye, and the difficulty of articulating his words, gave strong reason to suspect disease of the brain. On further examination, it was found that Mr. B. was totally blind of the left eye, and deaf of the left ear. There was considerable difficulty in clothing his ideas in language —and, indeed, there was also much confusion of thought, and some loss of memory. The pains in the head were excruciating, and generally produced vomiting once or twice a day. The pulse was extremely small, and very slow—sometimes irregular—the body was emaciated—the appetite almost gone—the bowels torpid. The pupil of the left eye was more dilated than that of the right, and little obedient to the light. There was no paralysis; but there was great debility of the limbs, with staggering, on attempting to walk. There were also cramps felt in different parts of the body, which, in a few weeks afterwards, amounted to convulsions resembling epileptic fits. After this examination, and receiving the account of the fall, no man could doubt that the disease was seated in the head, and that the sickness of stomach was merely symptomatic of the cerebral disease. The diagnosis and prognosis were stated to his wife, who was greatly shocked at the intelligence, the surgeon having assured her that it was merely disorder of the stomach. Counter-irritation in the neighbourhood of the head—leechings—cold to the scalp—and gentle aperient medicines gave a temporary relief, and entirely removed the gastric irritability, which never afterwards returned. But in a few weeks the cramps rose gradually to convulsions—the emaciation increased—the head-aches continued—the pulse became as feeble as a thread—and, on the 5th December, death put a period to his sufferings. During the two months preceding death, he was daily visited by Mr. Stevenson, surgeon, Edgeware Road, and the reporter only saw him once or twice a week.

On the 6th December the head was examined. On removing the calvarium the convolutions were seen to be much flattened and compressed, which prepared us for some tumour or hydrocephalic distention. The hemispheres were very firm, which firmness gradually decreased as we receded from the surface, and approached the corpus callosum. This part of the brain was very soft. There were four ounces of clear water in the ventricles. The septum lucidum was gelatinous, and there was a large perforation in the situation of the foramen of Monro. The iter ad infundibulum was so enlarged that the finger might be readily passed down to the sella turcica. All the parts in this neighbourhood were in a state of complete *ramollissement* The thalami nervorum were very much softened, and the tractus opticus was completely rotten. The optic nerves, especially the left one, were greatly wasted. On taking off the tentorium, a tumour composed of a cluster of hydatids, apparently with some tubercles intermixed, was seen closely adherent to the petrous portion of the left temporal bone, and pressing upon the left lobe of the cerebellum, as well as upon the medulla oblongata. It had completely annihilated the seventh pair of nerves on that side—and it had raised up the fifth pair of nerves, which were seen stretched tightly over the protuberant morbid mass. It was about the size of a small pullet's egg.

The loss of hearing is thus readily accounted for on the left side; but how was it that we had no distortion, paralysis, or loss of feeling in any part of the face, although the 7th pair of nerves was destroyed, and the fifth pair were greatly stretched and driven out of their natural situation by the tumour?

Considering the wanton manner in which the feelings of patients are sported with by some medical men—and reflecting on the foregoing sample of indiscrimination—nay, of positive error, arising from a preposterous adherence to a preconceived doctrine that blinds the eye to every object, we should feel it, in some degree, criminal to conceal such a monstrous mode of prescribing for diseases of the most dangerous or fatal nature, without giving one's self the least trouble to investigate the history, symptoms, or seat of the complaint. We hope this will meet the eye of the surgeon who so unceremoniously decided on the nature of the above-mentioned unfortunate gentleman's fatal malady. His feelings would not be enviable, we imagine, if he suffered himself to reflect, for a moment, on the terrible consequences which must often result from such a mode of carrying on the practice of medicine.

---

### THAMES WATER.

We understand that the Committee of Inquiry into the *purity* (God help us!) of the water supplied from the river to the inhabitants of this metropolis, is now sitting. We hope and trust they will do their duty. We have reason to believe that active exertions are making to enlist chemistry against cleanliness—and to persuade the town that, "what will not *poison* must *fatten*." We shall keep a very sharp look out upon the transactions of this Committee, and we promise to dissect the results of their inquiry with the sharpest critical instruments in our possession.

---

### SYMPATHETIC APOPLEXY.

Some people are as tenacious of life as worms, or as animals which we may divide into pieces without destroying vitality. A gentleman, who had long shewn symptoms of what Rostan and others would have termed "*Ramollessement du Cerveau*," fell down, the other day, in a fit of apoplexy, at the age of 68, and not the slightest impression was made by cupping, leeching, blisters, enemas, and all the means which a trio of physicians (including Dr. Warren) could suggest. Mapleson left the patient for dead, after taking four ounces of blood from the head; and he was apparently in articulo mortis, after 48 hours of general peralysis, total insensibility, stertorous breathing, glassy eyes, and "dead rattles" in the throat! The physicians parted—to meet no more—at least in that case. The ordinary physician took his leave at 12 o'clock at night, requesting to be informed in the morning, at what hour the patient died. No message having been sent, the physician called in the morning, and found, to his no small *surprise*, the patient at his breakfast, quite sensible, and with the full power of all his muscles!! The patient, soon after this, disgorged some pints of fetid bile, and had no return of apoplectic or paralytic symptoms. This is one of the many cases, where *irritation* of the chylopoietic nerves will simulate diseases of the most fatal character—and especially those of the brain and nervous system generally. Nothing is more common than partial pyralysis, particularly in children, from indigestible matters in the primæ viæ; and we are convinced that many of those apoplectic attacks, from which we see people quickly recover, without any remaining paralysis, are dependent on gastric or intestinal irritation. The foregoing case is a good example.

---

### LIBERTY OF THE PRESS.

We need hardly appeal to our readers, as to the fact of our having long and steadily resisted the calumnies and misrepresentations of the LANCET. For this we have been the constant theme of vituperation in that journal, for years past. Every one knows how Dr. Johnson has been abused, nick-named, and vilified in the Lancet; but few are aware that he is now threatened with the *fourth* law-suit, for daring to retaliate on a journal that is always bellowing forth about the LIBERTY OF THE PRESS, while abusing all, except the members of its own junto!

The first action was brought against Dr. J. as Editor of the MED.-CHIR. REVIEW, in the Summer of 1826. A similar action was brought against the PRINTER, for the same libel—and that for the sole purpose of *doubling* the law expenses, for Dr. Johnson never attempted to evade his responsibility. These two actions cost Dr. Johnson 700l. A third action was brought against Mr. Highley, the publisher, for having sold *to Mr. Wakley himself*, a single copy of the uncancelled journal, which had been accidentally returned to his shop, as a back number, from one of the houses in Paternoster Row! With a great deal of difficulty, Mr. Highley got the action compromised, by paying 32 pounds sterling, which Dr. Johnson considered himself bound, in honour, to repay. The fourth action is announced, for having, in our last Number, accused the LANCET, as a journal, of falsifying (through its reporters) the cases that occur in public hospitals—and of paying the highest price to those reporters that shew most ingenuity in garbling and distorting those cases occurring under physicians and surgeons, not of the JUNTO. Mr. Wakley takes all this to himself, though we have distinctly absolved him (and here repeat it) from these charges, and laid the blame on his reporters, his hireling writers—and on *some others* of the junto unknown or unseen. If Mr. Wakley will turn to page 247 of our last number, he will see, unless his vanity be much greater than his discernment, that we do not accuse *him* of bribing, with high fees, the falsifying reporters. "Will it be believed, that there are men, in this great metropolis, *who rank high in medical science*, but who are wicked enough to *expend considerable sums* in the reward of those who can invent the greatest, but, at the same time, the most plausible falsehoods, against the brightest characters in the profession." Is it possible that Mr. Wakley can be vain or shallow enough to suppose that we mean *him* in such passages? Could he get any man in London to say, in a court of justice, that *he* is the man alluded to? He knows full well he could not. In respect to the charges, then, against the LANCET, as a publication in which many are concerned, whose names do not appear, we retract not one iota of what we have advanced. If Mr. Wakley will still insist that he himself is *the* LANCET—a surgical INSTRUMENT, or a medical newspaper—why let him come into court in a shagreen case—or neatly folded up and *stamped*, price one shilling, and we swear by Apollo—

"Solem quis audeat falsum dicere."

Yea, by every God in Heaven—except MERCURY, the God of false reporters—and, therefore, perhaps one of the junto—that we will put a JUSTIFICATION on the record against the said LANCET, and verify our accusations by the greatest mass of evidence that ever came into the King's Bench or Common Pleas. Yes! and we will bring into court certain other personages, who, perchance, may wish themselves a thousand miles off on that fatal day!

"Give us the opportunity, (to use Mr. Wakley's own language, towards two of his EX-EDITORS) kind, modest Editor, of publicly extracting from these wretches, (REPORTERS) a *history of their own infamy*, and we will ever after call thee—FRIEND."

